# Hybridized Deep Learning Approach for Detecting Alzheimer’s Disease

**DOI:** 10.3390/biomedicines11010149

**Published:** 2023-01-06

**Authors:** Prasanalakshmi Balaji, Mousmi Ajay Chaurasia, Syeda Meraj Bilfaqih, Anandhavalli Muniasamy, Linda Elzubir Gasm Alsid

**Affiliations:** 1 College of Computer Science, King Khalid University, Abh 61421, Saudi Arabia; 2 Muffakham Jah College of Engineering and Technology, Hyderabad 500155, India

**Keywords:** Alzheimer, Adam’s optimization, convolutional neural network, long short-term memory

## Abstract

Alzheimer’s disease (AD) is mainly a neurodegenerative sickness. The primary characteristics are neuronal atrophy, amyloid deposition, and cognitive, behavioral, and psychiatric disorders. Numerous machine learning (ML) algorithms have been investigated and applied to AD identification over the past decades, emphasizing the subtle prodromal stage of mild cognitive impairment (MCI) to assess critical features that distinguish the disease’s early manifestation and instruction for early detection and treatment. Identifying early MCI (EMCI) remains challenging due to the difficulty in distinguishing patients with cognitive normality from those with MCI. As a result, most classification algorithms for these two groups perform poorly. This paper proposes a hybrid Deep Learning Approach for the early detection of Alzheimer’s disease. A method for early AD detection using multimodal imaging and Convolutional Neural Network with the Long Short-term memory algorithm combines magnetic resonance imaging (MRI), positron emission tomography (PET), and standard neuropsychological test scores. The proposed methodology updates the learning weights, and Adam’s optimization is used to increase accuracy. The system has an unparalleled accuracy of 98.5% in classifying cognitively normal controls from EMCI. These results imply that deep neural networks may be trained to automatically discover imaging biomarkers indicative of AD and use them to identify the illness accurately.

## 1. Introduction

The brain is commonly recognized as one of the body’s most essential organs. The brain controls and facilitates all activities and reactions that enable us to think and believe. Additionally, it helps us maintain healthy emotions and memories [1]. AD is a degenerative and incurable brain disease. When someone is diagnosed with Alzheimer’s disease [2], it advances slowly and kills memory cells, reducing the capacity of a person to think. It is a degenerative neurological disorder that results in the loss of function or even the death of neurons. Following Alzheimer’s diagnosis, the average lifespan is just four to eight years [3].

**Figure 1:**
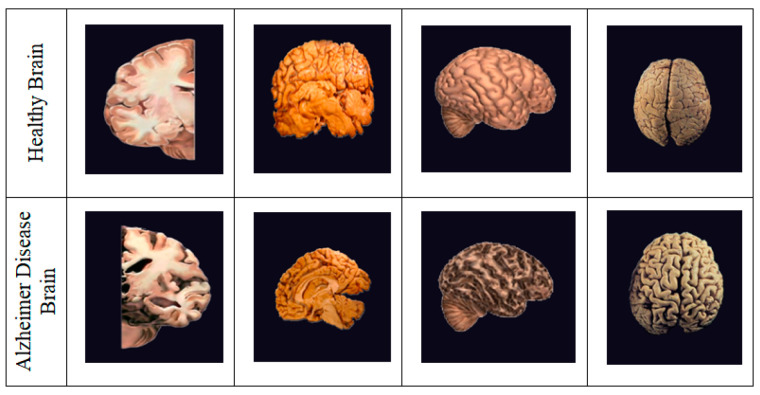
Healthy Brain vs. Alzheimer’s Diseased Brain. Compared to a healthy brain, the Alzheimer’s drain can be noted with shrinkage in size, less moisture, and enlarged ventricles.

According to recent research, 1 in every 85 people might be impacted by AD by 2050 [3]. It is critical to detect and treat AD patients in their early stages. There are numerous methods available for identifying and predicting this dementia, including MRI, PET, and CT (Computed Tomography) scans, as shown in Figure 1; however, the most often used neuroimaging modality for diagnosing AD patients is MRI [4,5]. Earlier research studies have used MRI-based classification techniques with Machine and Deep Learning algorithms [6]. This paper aims to develop the finest prediction and detection methods possible with the assistance of radiologists, clinicians, and carers to save time and aid patients suffering from this condition [7,8].

Alzhemier is the leading cause of dementia among the elderly. Dementia is defined as a decline in cognitive ability required for daily activities; AD accounts for 60–80% of dementia patients [9]. Plaques and tangles in the brain and cell damage and death indicate this condition. The doctor diagnosed her brain after her death and saw the formation of many clumps. These have been identified as the disease’s primary causal agents [10], which harmed the brain’s ability to communicate with the rest of the body.

Consequently, persons who suffer from this ailment have trouble doing daily duties such as driving and cooking. Early signs may include difficulty remembering names, misplacing important possessions, and planning [11]. AD is most severe during the middle stage, with symptoms including significant mood swings, disorientation, impulsivity, short attention span, and problems with object identification. The last stage is the most crippling [12].

The most noticeable symptoms include trouble communicating with others, increased vulnerability to infection, decreased judgment and sense of direction, short-term memory loss, and vision problems. According to a recent survey, around 50 million citizens worldwide suffer from AD [3]. It is known that the bulk of modern deep learning algorithms starts with a deep Convolutional Neural Network (CNN) model.

Recurrent neural networks, especially long short-term memory (LSTM), are effective models for processing sequential data [13,14]. DenseNet CNN architecture was adapted to extract the interslice features as it resolves the vanishing gradient problem, strengthens feature propagation, and reduces the number of parameters [15,16]. By considering all facts, a novel Deep Learning (DL) model that combines an LSTM on top of the bottleneck features extracted by initializing the weights of the pre-trained DenseNet architecture CNN that has already been trained on ImageNet is proposed. This hybrid model learns both intraslice and interslice features from the brain MRI data using CNN and LSTM.

This article discusses how to optimize the performance of a neural network for classifying sliced MRI data. The primary emphasis of this study is on the network’s use of various channels. In other words, the network will use all slices simultaneously rather than just one. The enhancement should come from common characteristics across numerous slices [17]. The Ant-Colony Optimization (ACO) [18] method was used to preprocess and denoise, while the Modified Fuzzy C-means (MFCM) algorithm performed clustering and feature extraction. The primary contributions and objectives of this manuscript may be summarized as follows:

- The fast feature extraction method for merging deep features collected from various parameters;
- Weight randomization reduces the breach among feature maps in the concatenation of fully connected layers;
- The hybrid model trained with CNN with LSTM model for the early diagnosis of AD;
- The development of the hybrid model meets the purpose of the research in detecting the adaptation of progressive MCI people to stable people;
- Additionally, this model achieves its secondary purpose by forecasting advancing individuals’ time-to-AD classes more accurately.

This study presents the multimodal DL approach for the early detection of AD (Multi-DL). A method for early detection of AD uses multimodal imaging and CNN with the LSTM algorithm. We generate MCI graphs utilizing a variety of data-gathering technologies and gender information. This solution successfully addresses the issue of individual variance, and the linkage of data between participants enhances the discriminability of various stages of MCI. The findings demonstrate that this strategy produced substantial prediction results after comprehensive validation.

DNN is a technique that is gaining traction among current approaches due to its efficacy in diagnosing AD. This work presents a novel hybrid DL-enhanced Adam optimizer for AD diagnosis using Python software.

Additionally, we utilized the expected rise in the cost of care for an AD patient. AD has surpassed cancer as the sixth biggest cause of death in the United States. Consequently, tailored computer-assisted methods are necessary for the precise and timely identification of this illness [19].

Alickovic et al. [1] presented a framework to identify AD using ML methods. Histograms are applied to change brain images to feature vectors that consist of pertinent “brain” features in the first stage. After this, ML techniques are applied for classification and identifying AD. The presented model has been elaborated using the current contribution that demonstrates a satisfactory level of performance. The experiment results prove that the RF classifier could discriminate AD subjects from control subjects. The proposed approach contains a histogram in the form of a feature extractor and the RF as its classifier with an accuracy of 85.77%.

Akbarpour et al. [2] presented classified statistical traits into many clusters using an unsupervised technique that segmented the data first—labeling individuals inside clusters and rearranging the images aided in obtaining their final picture. The outcome of this quantitative study demonstrated an optimal mix of fusion and segmentation, resulting in improved quantitative measures.

Cui et al. [3] provided a classification system for AD based on convolutional and recurrent neural networks (RNNs). To begin, CNN was developed to learn spatial elements in MR images for categorization. The RNN and cascaded Bidirectional Gated Recurrent Unit (BGRU) layers were developed utilizing CNN outputs at different periods to extract longitudinal characteristics in AD classification. Rather than extracting features individually, the strategy recommended learning spatial and longitudinal characteristics together to optimize performance. Additionally, the suggested technique would use an RNN to construct a longitudinal study using imaging data collected at various periods in time. The findings demonstrated that the suggested technique produced a higher accuracy of around 91.33% when comparing AD to NC and 71.71% when comparing Progressive Mild Cognitive Impairment (pMCI) to Stable Mild cognitive impairment (sMCI).

Kowalski and Łukasik [10] contrasted the KH optimization algorithm employed for ANN learning and other heuristics and traditional methods. The ANN technique was verified for classification. Several benchmark examples were obtained from the UCI ML Repository and applied along with the sum of Square Error and Classification Error as evaluation parameters. A conclusion was made that implementing the KH showed great performance for the criteria mentioned earlier and in the time needed for ANN training.

Liu et al. [11] proposed multitasking feature selection, a technique to preserve inter-modality and enforce feature sparseness in each modality. As soon as features are selected, the multi-kernel SVM is employed to incorporate the features selected through a classification modality. This method was evaluated using the MRI images of the subjects and the baseline Positron Emission Tomography (PET) duly obtained from the database of the ADNI.

Yue et al. [16] proposed another novel Voxel-based Hierarchical Feature Extraction (VHFE) technique to diagnose AD at an early stage. It parcellates the entire brain into 90 different ROIs using a template Automated Anatomical Labeling (AAL). For splitting uninformative data, informative voxels for each ROI using a baseline of values were employed and arranged into one single vector. The first stage of these features was chosen, and the brain feature maps for every subject using voxels were fed to the Convolutional Neural Networks (CNN). Lastly, to validate the method’s effectiveness, the authors tested them on a subset of an ADNI database. The testing results proved the robustness of the method compared to other methods.

Zheng et al. [17] proposed another new presentation of a summary of all current protocols for the automatic detection of dementia from their actual perspective and patterns in classification. Since the protocols consist of feature extraction in all these cases, three different types of techniques were offered. The vertex, voxel, including RoI-based and group classification algorithms: the SVM, Artificial Neural Networks (ANN), Linear Discriminant Analysis (LDA), and Bayes classifier. The final execution of these classifiers proved that there were many protocols to distinguish AD from Head Circumference (HC) aside from the accuracy.

Shankar et al. [20] proposed an approach for AD and Brain Image Analysis (BIA). In the initial stage, unwanted regions in the images are removed. After this, several features, like texture scale-invariants, transform, and histograms were extracted. Group Grey Wolf Optimization (GGWO) techniques increase the detection performance with a decision tree, CNN, and KNN classifiers. They are used for identifying a reduced set of features without reducing performance. This approach obtained 96.23% accuracy in AD detection compared to other competitive schemes.

Problem formulation is taken as:

- The FCM algorithm’s distance measures have unequal clusters of lower accuracy, so we move to the MFCM algorithm for image segmentation and clustering to gain higher accuracy;
- The training data and the model utilizing it seek equal importance in prediction; hence, we implemented a hybrid training model as CNN with LSTM for higher training and perfect prediction;
- Adam’s does not converge to an optimal solution in some areas, and it can suffer a weight decay problem, so we proposed an improved Adam’s optimizer. Then, the early AD prediction is much easier.

The rest of this article is organized in the same manner. The suggested model is shown in Section 2. Section 3 summarizes the study’s findings. Finally, Section 4 outlines the conclusion and future work.

## 2. Materials and Methods

**Figure 2:**
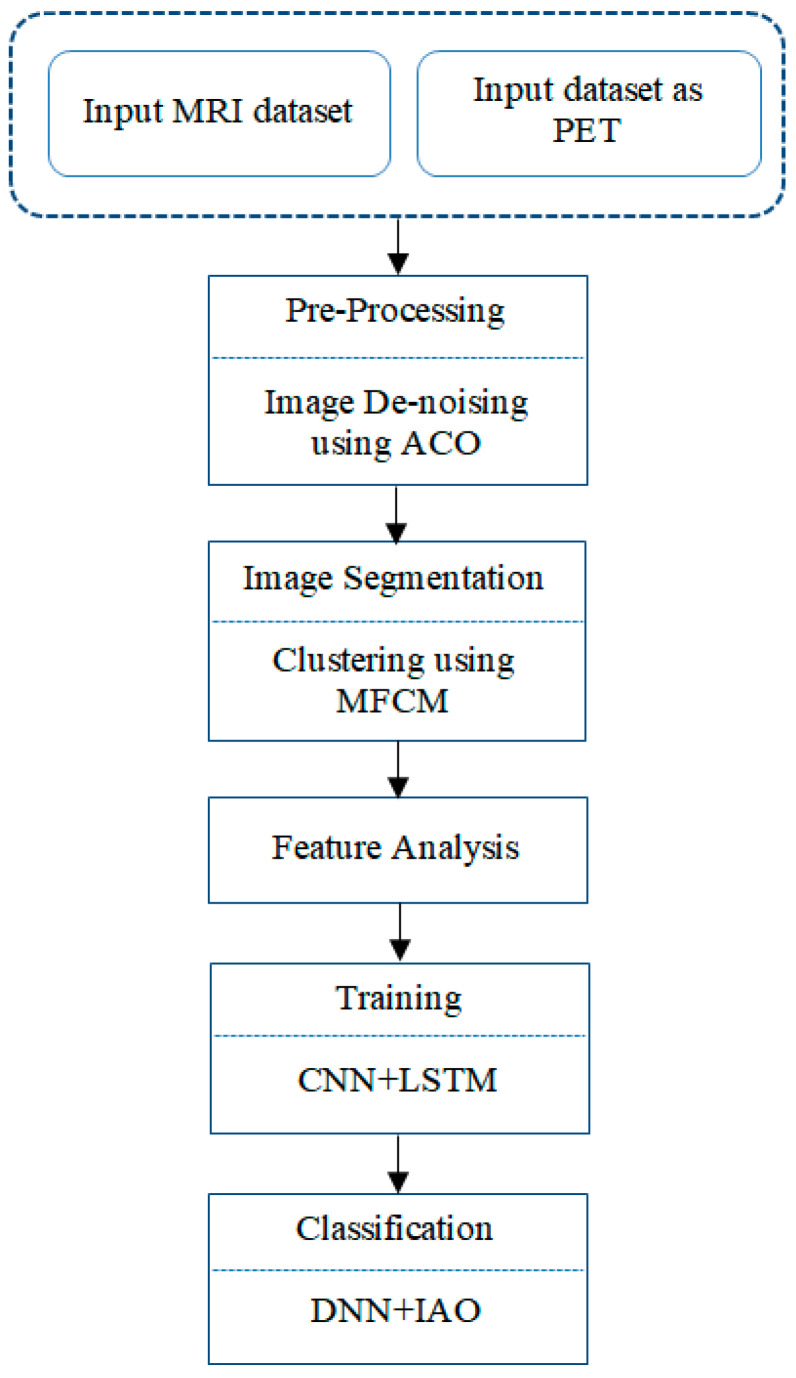
Flow chart for Multi-DL showing the stages of the proposed system involving conventional steps followed: Pre-processing; image segmentation; feature analysis; training; classification.

The hybrid system’s primary objective is to detect AD early. Brain atrophy is a crucial factor in the diagnosis of AD. The MRI dataset values are preprocessing and noise removal using the ACO algorithm. The MFCM image segmentation technique identifies brain atrophy, and the atrophy of the cerebral cavity was verified using image gradients. A gradient in a photograph refers to a variation in the image’s color or intensity. The combination of the CNN+LSTM algorithm has been used to train the dataset for up to 20 epochs. The optimization and classification procedure has been done by the IAO method. The previous method employs a straightforward procedure and a modest level of visual complexity. The proposed technology eliminates early detection without causing brain damage and completes 98.5% of the indicated procedures. Deep Learning methods classify the patient as stable, first-stage AD, second-stage AD, or moderate cognitive dysfunction. The proposed system contributes to the medical imaging field, and it showed higher accuracy performance when compared to other existing ones. The early detection and classification of AD are more accurate in the proposed system, as shown in Figure 2.

### 2.1. Dataset Collection

**Figure 3:**
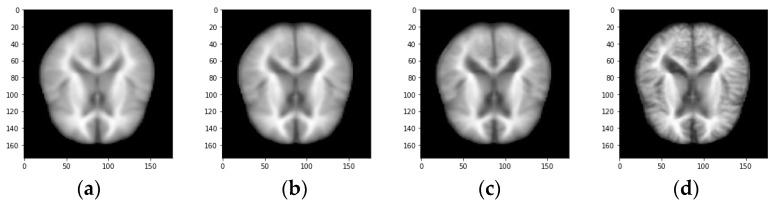
Sample MRI image demented categories. (**a**) Non-demented image (**b**) Very mild demented image (**c**) Mild demented (**d**) Moderate demented.

**Figure 4:**
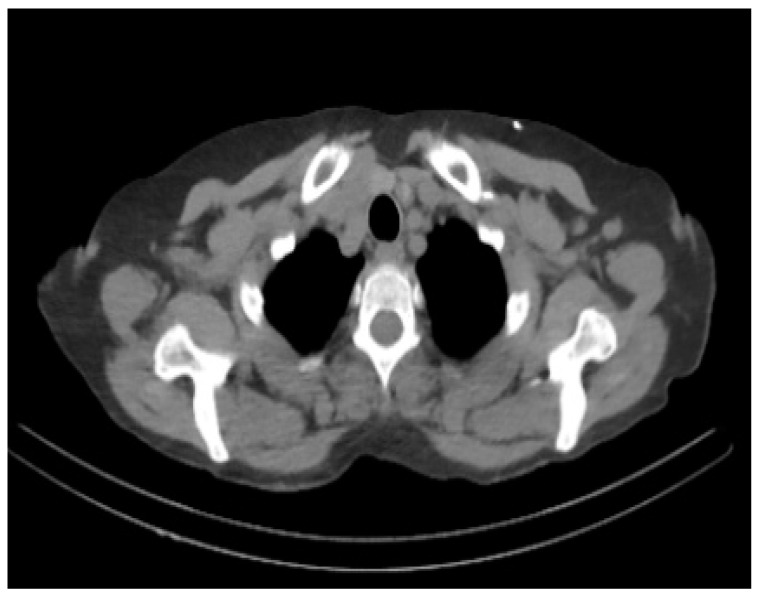
Sample PET scan image showing an Alzheimer’s affected brain. Noted is the size of the enlarged ventricles.

The proposed work aims at the early detection of AD. The data sets play an important role as the training data is needed to train the model for performing the needed actions. Most of the data in the data set used for training the model and data set is also separated into test data. The proposed system executes the algorithm on AD-related datasets obtained from Kaggle with 512 MRI images and the Munich database with 112 PET images; Figure 3 and Figure 4 show the sample image data.

The Non-demented, average, very mild, mild demented, and moderate demented MRI images in Figure 2 and Figure 3 show the sample PET scan image.

### 2.2. Pre-Processing Stage

The preprocessing phase improves the image’s accuracy and lowers noise. Because brain images are more sensitive than other medical imaging, there must be as little noise as possible while maintaining optimum visibility. The image conversion from color to grayscale, resizing the image, reshaping the image, sharpening the image, etc. are taken up in preprocessing the image 

Techniques for preprocessing MRI images increase the identification of suspicious regions. The preprocessing and augmentation technique comprises two steps: first, a tracking algorithm eliminates film artifacts from the MRI, such as labels and X-ray markers. Second, high-frequency components are deleted using the ACO approach. Pre-processing with tracking functions that include noise reduction is often required before doing detailed data evaluation and extraction and is commonly categorized as a radiometric or geometric enhancement.

Based on the route’s pheromone content and the interpretation of the heuristics, high-frequency components are selected. This is how nodes are built in MRI. During the building process, an ant *k* uses a probabilistic action selection rule to decide which node to move to next depending on the chance that ant *k* will choose from the current node *i* to the next node:

(1) $$p_{ij}^{k}\frac{{\left[T_{ij}\right]}^{\alpha }{\left[n_{ij}\right]}^{\beta }}{\sum_{N_{i}}^{k}[T_{ij}{]}^{\alpha }[n_{ij}{]}^{\beta }}if\;j\in N_{i}^{k}$$

where $T_{ij}$ illustrates the concentration of the arc from node *i* to node *j*, $N_{i}^{k}$ Illustrates the neighborhood nodes for an exact ant *k*, provided it is on node *i*. The constants α and *β* indicate the pressure of the content and heuristic equally.

### 2.3. Image Segmentation and Clustering

The area of interest’s orchestrated location is determined, and the chosen region is planted and zoomed in. This image is used to modify the pixel’s intensity. Pixels are referred to as white or black in the picture, depending on their brightness. The white area illustrates living tissue, whereas the black area illustrates dead tissue. The number of white and black pixels is computed, and if the black pixel number is very low, the patient is deemed healthy. According to the black pixel ratio, the patient has a slight learning impairment, AD, or is healthy. Thresholding is a widely used technique for picture segmentation. Grayscale or pixel power segmentation is achieved in this image segmentation technique. It is two-tier thresholding for classifying pixels into two groups based on the intensity of the image pixels.

Given, *X* = {*x*_1_, ….*x_n_*}⊂ $R^{p}$, the FCM partitions *X* into *c* fuzzy subsets by reducing the following objective function

(2) $$J\left(U,V\right)=\sum_{i=1}^{c}\sum_{k=1}^{n}U_{i}^{m}\;\|X-V_{i}{\|}^{2}$$

where *c* is the number of clusters, which is favored in this study as a predetermined significance level, *n* denotes the number of data points, *U_ik_* is the membership of *X_k_* in class *t*, satisfying $\overset{c}{\underset{i=1}{\sum }}u_{ik}=1,m$ the quantity controls clustering fuzziness, and *v* the set of cluster centers or prototype (vi€ Rp). A well-known alternating iterative algorithm minimizes the function *J_m_*.

Now consider the proposed MFCM algorithm. Describe a nonlinear map as $\emptyset x\;\to \;\emptyset \left(x\right)\in F$, where *x* € *X*, *X* represents the data space, and *F* represents the transformed feature space with higher, even infinite dimension. MFCM reduces the following objective function:

(3) $$J\left(U,V\right)=\sum_{i=1}^{c}\sum_{k=1}^{n}Ui^{m}\;\|\emptyset (X)-\emptyset \left(Vi\right){\|}^{2}$$

(4) $$||\;\emptyset \left(X_{K}\right)-\emptyset \left(V_{i}\right)|{|}^{2}=K\;(X_{K},X_{K})+K\;(V_{i},V_{i})-2K\;\left(X_{K},V_{i}\right)$$

where K(x,y) = $\emptyset {\left(x\right)}^{T}\emptyset \left(y\right)\;$ is an inner product kernel function. If we adopt the function as a kernel function, i.e.,

K(x,y) = exp ($-{\left\|x-y\right\|}^{2}/\sigma^{2})$, then K(x,x) = 1, according to Equation (5), Equation (6) can be rewritten as:

(5) $$J\left(U,\;V\right)=2\sum_{i=1}^{c}\sum_{k=1}^{n}U_{i}^{m}\;(1-K(X_{k},V_{i}))$$

Minimizing Equation (6) under the constraint of $\mu_{ik}$, we have:

(6) $$\mu_{ik}=\frac{{\left(1/\left(1-K\left(x_{K},v_{i}\right)\right)\right)}^{\frac{1}{m-1}}}{\sum_{j=1}^{c}{\left(1/(1-K\left(x_{K},v_{i}\right))\right)}^{\frac{1}{m-1}}}$$

(7) $$V_{i}=\frac{\sum_{k=1}^{n}U_{ik}^{m}K\left(X_{k},V_{i}\right)X_{k}}{\sum_{k=1}^{A}U_{ik}^{m}K\left(X_{k},V_{i}\right)}$$

Here we presently use the function for effortlessness. Equivalent modifications will be found in Equations (8) and (9) if we use other kernel functions. Equation (3) can be viewed as a kernel-induced new metric in the information space, defined as the subsequent equations:

(8) $$d(x,y)=||\;\emptyset \;(x_{k})-\emptyset \;(v_{i})\;||=\sqrt{2\left(1-k\left(x,y\right)\right)}$$

It can be proved that d(x,y) defined in Equation (8) is a metric in the innovative space in the case *k*(*x*,*y*) takes as the function.

According to Equation (8), the information point $x_{k}$ is endowed with an extra weight k($x_{k}$,$v_{i})$, which measures the correspondence between $x_{k}$ and $v_{i}$, and when $x_{k}$, is an outlier, i.e., $x_{k}$ is far from further data points, then k($x_{k}$,$v_{i})$ will be very little. As a result, the weighted aggregate of data points will be more balanced.

Clustering methods are uncontrolled segmentation techniques that separate an image into comparable intensity pixel/voxel clusters without training images. Clustering algorithms use accessible picture data for self-practice. The segmentation and planning were conducted in two steps: data clustering and tissue sort estimation features.

(9) $$j_{m}=\sum_{i=1}^{c}\sum_{j=1}^{N}u_{ijD_{ij}}^{m}$$

Each region is lit independently, depending on the cluster’s position. It is processed at a high rate of noise cancellation with poor homogeneity figures and a high rate of morphologic image filtering.

The points, edges, objects, etc., are the most common features seen in an image. A feature may be thought of as a piece of information about the content of a picture; the information shows whether or not a certain portion of the image has specific attributes. The classification of the feature details based on generated and formulated information is registered with the binary texture pattern.

### 2.4. Training Procedure

**Figure 5:**
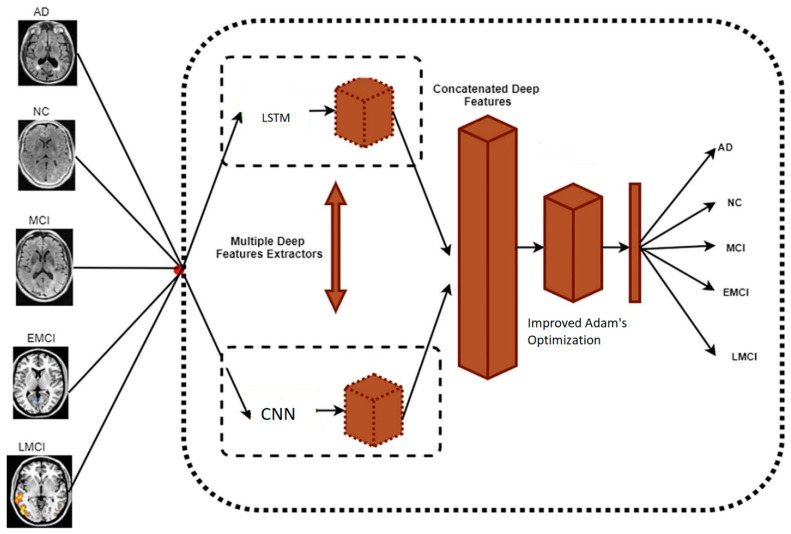
The hybrid framework model shows the multiple deep feature extractors by LSTM and CNN and is Adam optimized to classify the input image as Alzheimer affected or non-demented.

The proposed hybrid framework is displayed in Figure 5. The input image is trained with CNN with LSTM and features.

a. Convolutional Neural Network

Initially, CNN was employed to handle difficulties with picture recognition. It is currently limited to photographic and video images and is used to represent time-series signals such as audio and text. The advantage of CNN is the localized convolutional weight-sharing structure, which allows for large reductions in the size of the neural network, avoidance of overfitting, and reduction in model complexity. By using spatial structural linkages, CNNs may minimize the number of parameters to learn. As a consequence, backpropagation algorithms’ efficiency during training may be boosted. The first convolutional layer of CNN receives input directly from the pixels of the picture. Each convolution operation takes a limited number of pictures, performs convolutional modifications, and transfers the data to the back network. Each convolutional layer extracts the data’s most efficient characteristics. CNN may be defined using its graph-theoretic formulation. The convolution filter’s parameters are shared across all sites in the network layer. The feature map calculates the spatial feature by computing the weighted average of the center pixel and the neighboring pixels. Our model defines the network layer as follows:

(10) $$H^{l+1}=f(H^{l},A)$$

(11) $$f\left(H^{l}A\right)=\sigma \left(AH^{l}W^{l}\right)$$

where *W^l^* denotes the weight matrix of the *l*th layer neural network and is a non-linear active function. As a result, we may use LSTM with CNN to replace the kernel with an optimizable graphical convolutional network model.

b. LSTM

It is possible to associate prior data with the current task using the LSTM, a recurrent neural network. A fully connected and activated LSTM network is then created to capture the temporal relationship between features and the next stage of AD. The LSTM technique avoids the problem of long-term dependence via a series of repeated neural network modules. Each patient’s extracted features are combined with MRI data previously entered into the LSTM cell. The output is fed into a single fully connected layer to convert the LSTM’s multidimensional output to one with a probability between 0 and 1. This network will be trained to forecast a patient’s chance of getting Alzheimer’s disease over the next five years, balancing that probability with a loss function that collects diagnoses for specific individuals over time.

(12) $$f_{t}=\sigma \left(W_{f}.\left[h_{t-1},\;x_{t}\right]+b_{f}\right)$$

(13) $$i=\sigma \left(W_{f}.\left[h_{t-1},\;x_{t}\right]+b\right)$$

(14) $$h_{t}=o_{t}*\tan h\left(C_{t}\right)$$

The worth of *o_t_* in Equation (14) decides which element of the unit will be the productivity. The new cell state *C_t_* is multiplied by *o_t_*, and the function tanh is chosen to obtain *ht* in Equation (9), the input of parts *ij*. The LSTM can learn long-term dependencies; a model expressing the course of AD is developed using the LSTM.

Next, combine feature encoding from CNN with feature encoding from past images for the patient. New memory *c*^(*t*)^ is generated by using the input data *x*^(*t*)^ and the precedent hidden state *h*^(*t*−1)^ (note that *W* and *U* refer to weights and parameters.)

(15) $$c^{t}=\tan h(w^{c}x^{t}+U^{c}h^{\left(t-1\right)})$$

Using the input data and the previous concealed state, the input gate generates a result *i*^(*t*)^ to gate the new memory, determining what information is valuable and should be remembered.

c. Improved Adam’s Optimization

The suggested approach includes several data fields used to calculate the average data for the categorized segment. Several criteria must be completed before Medicare reimburses a PET scan for people living with dementia. Frontotemporal dementia is suspected due to the start, clinical presentation, and curve of cognitive impairment. A full clinical assessment is required, including a physical and mental status examination that demonstrates a cognitive deterioration lasting at least six months and neuropsychological testing, laboratory tests, and structural imaging.

The improved Adam approach improves the hidden layers and target function, resulting in a considerable reduction in error rates. The corrected Adam optimizer does not require stationary targets based on sparse gradients. The model’s parameters are: Softmax learning rate is 50, sparsity percentage is 0, scale data is fake, L2 weight regularization is 0.005, hidden layers are 100, and sparsity regularization is 5. The maximum iteration is 10. Softmax’s learning rate is 50. The maximum number of iterations is 10, and the SAE learning rate is 50.

## 3. Results and Discussions

The proposed deep learning algorithms are assessed using a test dataset (n = 512 MRI scans and 112 PET images) that comprises 60% of the total data. Separate from the training dataset, the test dataset is used to assess the model’s generalizability and ability to make predictions on previously unknown data. Stratification is used to balance the classes included within the two databases. The hybridized model is qualified and evaluated repeatedly (n = 20 epochs) to get the outcome shown in Table 1.

**Figure 6:**
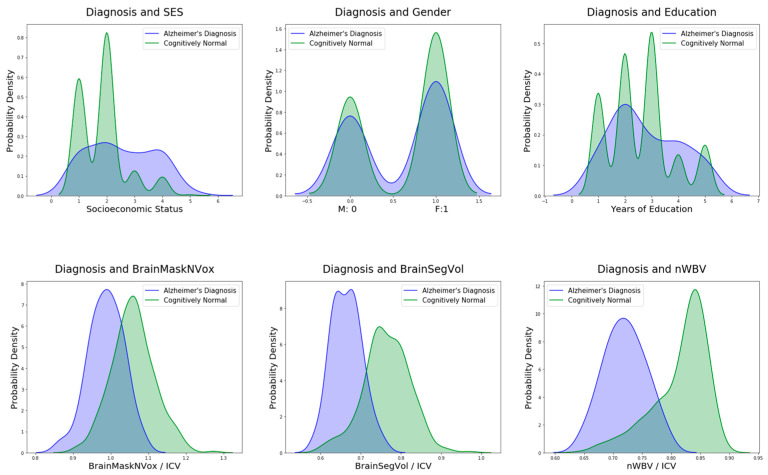
AD diagnosis of various parameters based on the database values taken up for each individual before imaging.

The proposed model obtains a standard accuracy of 92.8% and 98.5% on the test dataset. When the receiver operating characteristic (ROC) area under the curve (AUC) is evaluated, the model performs better, with an average value of 0.80 to 0.83. When the boxplots in Figure 6 are compared, the accuracy and ROC for conversion and risk classification are comparable. On the other hand, specificity has a far greater conversion variety than risk, with values as low as 7.3% and as high as 92.3%. In general, the model outperforms random classification when identifying patients with progressive MCI and time to admission. The proposed hybrid model is 19.8% more accurate than random chance differentiating pMCI from sMCI patients (50%). Compared to random chance, the model correctly classifies patients with pMCI into their proper time-to-admission group by 33.9%.

This research aims to develop a convolutional neural network–LSTM hybrid capable of discriminating moderately cognitively impaired individuals who progress to AD from those who remain stable. Additionally, the proposed model anticipates the period required for AD conversion, classifying those who indicate that conversion will occur within 24 months (large risk) or greater (little risk) and those who have no risk (sMCI). 

The data gathered are baseline measures taken at an individual’s first visit. Cross-sectional data replicate the circumstances of an individual’s first healthcare visit.

The proposed hybrid model is constructed using a range of contemporary high-performance techniques. The literature review summarizes recent advancements in early AD prediction research in terms of preprocessing and Deep Learning approaches. Existing research has produced a variety of methods for predicting pMCI vs. sMCI, many of which include an image listing of the MRI information in their preprocessing pipeline. Additionally, effective results surpassing 80% in cross-validation accuracy and AUC may be ascribed to domain learning to extract the most valuable auxiliary features from a related domain, i.e., AD vs. cognitively normal categorization. Each linked experiment improved the validation accuracy when domain learning was utilized.

**Figure 7:**
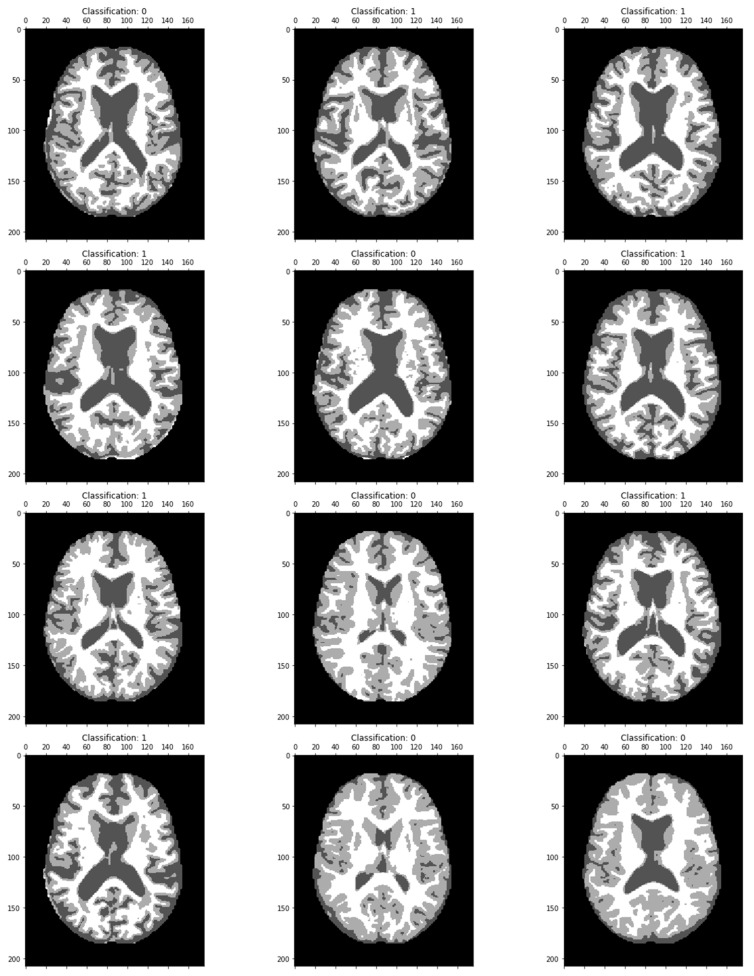
MRI image classification results showing the classification value as 0, confirming the absence of Alzheimer’s disease, and Classification 1 shows the brain image of an Alzheimer-affected person.

The MRI image classified results are displayed in Figure 7. Dementia has been classified in each image as a particular region.

**Figure 8:**
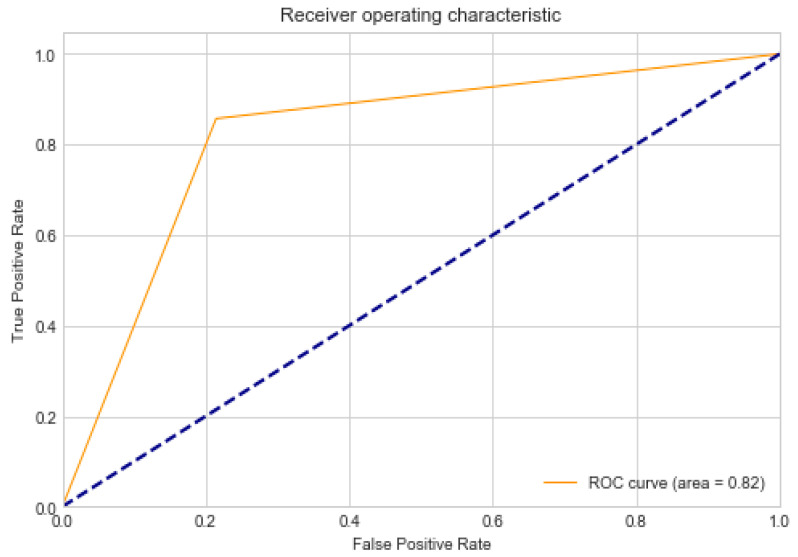
ROC curve comparing the True Positive Rate and False Positive Rate with the value of 0.82.

The proposed ROC curve value is plotted, and the True Positive and False Positive is represented in Figure 8.

**Figure 9:**
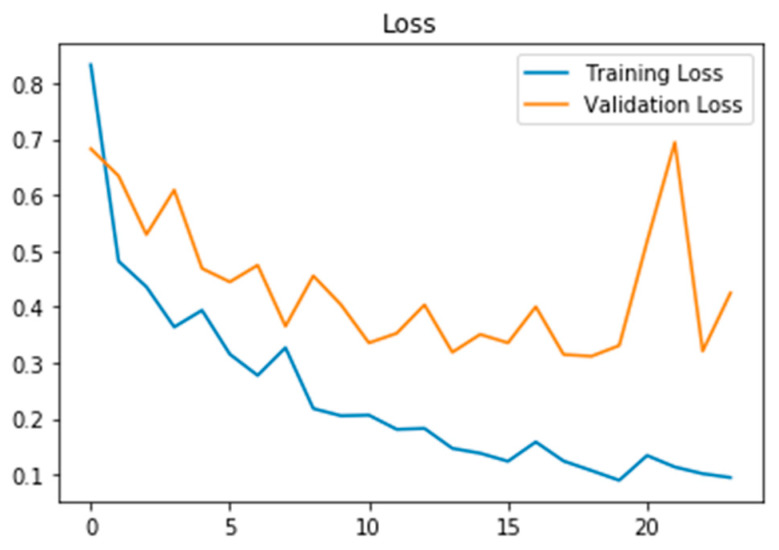
Training and validation loss for the first 20 epochs with a stable difference in training and validation loss showing a good fit curve.

**Figure 10:**
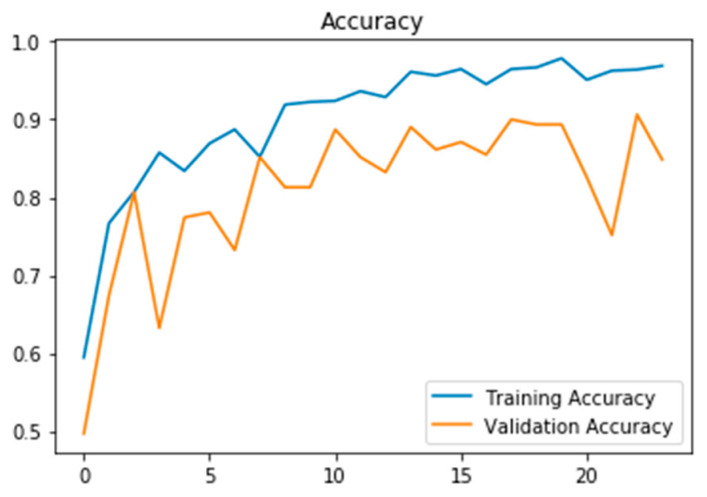
Training and validation accuracy showing the least gap between the accuracy values in training and validation sessions proves a good fit model.

Figure 9 depicts the training loss and validation loss after 20 epochs, whereas Figure 10 depicts the training and validation accuracy after 20 epochs.

**Figure 11:**
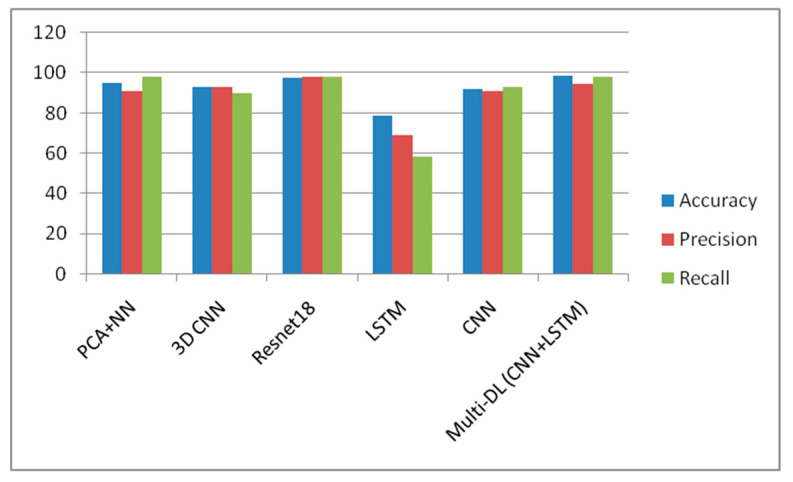
Accuracy comparison chart for various methods showing a better performance in the proposed CNN, LSTM combination.

The proposed model has been compared with various methods like PCA, CNN, Resnet18, and DCNN. Table 2 illustrates the accuracy, precision, and recall values and the comparison chart in Figure 11.

Concerning the paper’s aims and objectives, the development of a hybrid model meets the research objectives since it can identify the conversion of stable MCI persons to advancing MCI individuals. Additionally, the model accomplishes its secondary objective of anticipating developing individuals’ time in AD class with greater accuracy. However, the findings of Multi-validation DL show that extra effort is required to build the model’s architecture and optimize its hyperparameters. Due to the limited amount of data and the pipeline’s conformity to current standards, the only way to improve feature extraction and performance is to alter these steps. Maximizing the utilization of existing data in a domain with little data is vital. Domain learning may be one strategy for improving the identification and prediction of advancing MCI and its progression to AD. According to a literature survey on current approaches, domain learning has shown a favorable effect on model performance and the number of articles that employ the method. While training the weights of the model to detect the characteristics of auxiliary AD in addition to non-AD classes will not improve the model’s presentation of the core issue, it will speed the convergence and hence reduce the training time.

Segmentation of the brain is another approach that may enhance performance. Brain segmentation (temporal, parietal, prefrontal, and occipital lobes) allows parallel three-dimensional convolutional layers to extract more precise information from these areas, condensing the complicated feature space. A more compact feature area should make it easier to find useful features.

## 4. Conclusions

Recent advances in biomedical engineering have elevated the study and interpretation of medical pictures to critical research fields. One of the reasons for this advancement in medical image analysis is the use of DL. Classification by DL has been mostly employed during the last year. Automatic early detection of AD is an AI-based technology that satisfies physicians’ major aims. Thus, an automated framework and classification system for AD based on MRI images are crucial for the early detection of AD patients. A hybrid approach is proposed for predicting early AD using MRI data. The two datasets [21] used in this study were 512 MRI and 112 PET pictures for training. ACO performed the noise reduction process, MFCM performed the picture segmentation process, and CNN and LSTM performed the training process. Finally, categorization was accomplished via deep neural networks, which improved Adam’s optimization strategy. The suggested technique is compared to several other methods. The accuracy level of the proposed model has been increased to 98.5%. Among the findings obtained with various epoch sizes, a significant result was achieved with an epoch size of 20 and an accuracy rate of 98.5%.

In the future, we anticipate and encourage more effort. Thus, the outcome might be enhanced by using a deep CNN with transfer learning methods.

## Figures and Tables

**Table 1:** 20-epochs data with training loss, test accuracy.

Epoch	Training Loss	Test Accuracy	Epoch	Training Loss	Test Accuracy
1	21.77	92.56	11	12.23	97.12
2	21.28	93.41	12	11.45	97.86
3	19.20	94.54	13	10.56	97.92
4	18.34	94.78	14	9.89	98.11
5	17.34	94.89	15	9.12	98.14
6	16.32	95.12	16	7.89	98.34
7	15.77	95.67	17	6.89	98.37
8	14.67	95.89	18	6.78	98.41
9	13.56	96.78	19	5.89	98.45
10	13.12	96.89	20	5.67	98.51

**Table 2:** Accuracy, precision, recall.

Model	Accuracy	Precision	Recall
PCA+NN	95	91	98
3D CNN	93	93.18	90
Resnet18	97.88	98.10	97.89
LSTM	78.72	68.96	58.66
CNN	92	91	93
Hybrid (CNN+LSTM)	98.5	94.8	98

## Data Availability

Online data sources from Kaggle are used throughout the study. https://www.kaggle.com/tourist55/alzheimers-dataset-4-class-of-images (accessed on 16 July 2022) and https://deepblue.lib.umich.edu/data/concern/data_sets/st74cq55j?locale=en (accessed on 16 July 2022).
